# Work Intensity, Low-Grade Inflammation, and Oxidative Status: A Comparison between Office and Slaughterhouse Workers

**DOI:** 10.1155/2018/2737563

**Published:** 2018-04-18

**Authors:** Sieglinde Zelzer, Franz Tatzber, Markus Herrmann, Willibald Wonisch, Stefan Rinnerhofer, Michael Kundi, Barbara Obermayer-Pietsch, Tobias Niedrist, Gerhard Cvirn, Georg Wultsch, Harald Mangge

**Affiliations:** ^1^Clinical Institute of Medical and Chemical Laboratory Diagnostics, Medical University of Graz, Auenbruggerplatz 29, 8036 Graz, Austria; ^2^Center of Molecular Medicine, Institute of Pathophysiology and Immunology, Medical University of Graz, Heinrichstrasse 31a, 8010 Graz, Austria; ^3^Institute of Physiological Chemistry, Center for Physiological Medicine, Medical University of Graz, Stiftingtalstrasse 6 M1/D/3, 8036 Graz, Austria; ^4^Exercise Physiology, Training and Training Therapy Research Group, Institute of Sports Science, University of Graz, Mozartgasse 14, 8010 Graz, Austria; ^5^Department of Environmental Health, Center for Public Health, Medical University Vienna, Kinderspitalgasse 15, 1090 Vienna, Austria; ^6^Division of Endocrinology and Diabetology, Department of Internal Medicine, Medical University of Graz, Auenbruggerplatz 15, 8036 Graz, Austria; ^7^Arbeitsmedizinisches Institut, Graz, Herrgottwiesgasse 149, 8055 Graz, Austria

## Abstract

Limited knowledge exists about the impact of physical workload on oxidative stress in different occupational categories. Thus, we aimed to investigate the oxidative and inflammatory status in employees with different physical workloads. We enrolled a total of 79 male subjects, 27 office workers (mean age 38.8 ± 9.1 years) and 52 heavy workers, in a slaughterhouse (mean age 40.8 ± 8.2 years). Fasting blood was drawn from an antecubital vein in the morning of the midweek before an 8-hour or 12-hour work shift. The antioxidative capacity was assessed measuring total antioxidant capacity (TAC), uric acid, total polyphenols (PPm), and endogenous peroxidase activity (EPA). Total peroxides (TOC), malondialdehyde (MDA), and myeloperoxidase (MPO) were analyzed as prooxidative biomarkers, and an oxidative stress index (OSI) was calculated. In addition, hsCRP, interleukin-6 (IL-6), MDA-LDL IgM antibodies, galectin-3, adrenocorticotropic hormone (ACTH), and the brain-derived neurotrophic factor (BDNF) were measured as biomarkers of chronic systemic inflammation and emotional stress. TOC (*p* = 0.032), TAC (*p* < 0.001), ACTH (*p* < 0.001), OSI (*p* = 0.011), and hsCRP (*p* = 0.019) were significantly increased in the heavy workers group, while EPA, BDNF (*p* < 0.001), and polyphenols (*p* = 0.004) were significantly higher in office workers. Comparison between 8 and 12 h shifts showed a worse psychological condition in heavy workers with increased levels for hsCRP (*p* = 0.001) and reduced concentration of BDNF (*p* = 0.012) compared to office workers. Oxidative stress and inflammation are induced in heavy workers and are particularly pronounced during long working hours, that is, 12-hour versus 8-hour shifts.

## 1. Introduction

Modern life-style, that is, physical inactivity and fast food as well as occupational and environmental conditions, may induce oxidative stress. Different types of stress can be distinguished at the cellular and tissue level—namely, photooxidative stress, drug-dependent oxidative stress, metabolic oxidative stress, environmental oxidative stress, and nitrosative stress [1].

Reactive oxygen species (ROS) are endogenously generated, among others, in the respiratory chain. Hence, metabolic activity increases ROS production. These species react with biological molecules like lipids, carbohydrates, proteins, and even DNA, which are associated with the pathogenesis of degenerative diseases. Oxidative stress (OS) is associated with chronic inflammation, with a potential impact on diabetes mellitus, atherosclerosis, and cardiovascular and neurodegenerative diseases [2–5]. An increased consumption of oxygen during physical exercise also increases ROS production leading to oxidative stress and lipid peroxidation in athletes [6–8]. Nevertheless, increased ROS production during sports is also beneficial because it stimulates the antioxidative system [9]. Moreover, OS is a key factor during aging [10] together with other factors like deregulated autophagy, mitochondrial dysfunction, and telomere shortening [11]. Besides its involvement in the physiologic process of aging, OS appears to play an important role in the pathophysiology of several occupational diseases [12, 13]. Common problems in night and shift workers, such as fatigue, sleep problems, anxiety, difficulties in maintaining regular life-styles, and reduced recovery times, represent an increased health risk due to physiological exhaustion and a decreased capacity for regulation [14].

Nevertheless, there is limited knowledge about the impact of physical workload on OS in different occupational groups. Heavy workers often suffer from excessive workload and lack of social support. Shift work with extended working hours might negatively affect the psychological status of employees and reduce their motivation. It can be hypothesized that high physical and emotional stress in heavy workers is associated with increased OS and inflammation.

The present study aimed at comparing the oxidative and inflammatory status between office workers and heavy workers with a particular focus on the biochemical effect of extended working hours (8- to 12-hour shifts).

## 2. Materials and Methods

### 2.1. Study Population

We enrolled 79 healthy male volunteers between 18 and 65 years at their workplace. Thereof, 27 employees were office workers (age 38.8 ± 9.1 years) and 52 heavy workers in a slaughterhouse (age 40.8 ± 8.2 years). Exclusion criteria were infections, for example, flu-like infection, chronic diseases, and certified reduced work capacity due to illness. The study was approved by the ethics committee of the Medical University of Graz (EK number 26-488 ex 13/14) and conducted in compliance with guidelines for human studies as described in the Helsinki Declaration of 1975, revised in 1996. Written inform consent was obtained from all study participants.

### 2.2. Laboratory Analysis

#### 2.2.1. Blood Sampling

Blood was drawn from an antecubital vein between 6:00 a.m. and 6:30 a.m., before an 8-hour work shift from 79 workers (27 office and 52 heavy workers). In a subgroup of 26 office workers and 8 heavy workers, we investigated the effects of twelve hours of work. Blood sampling was performed in the midweek, Wednesdays or Thursdays. Samples were immediately transferred on ice to the Lab within two hours, centrifuged, and stored at −80°C until use (6 to 13 months).

#### 2.2.2. Inflammatory Parameters

High-sensitivity C-reactive protein (hsCRP) and interleukin 6 (IL-6) were determined on a COBAS® 8000 analyzer with turbidimetric and electrochemiluminescence immunoassays (ECLIA), respectively, from Roche Diagnostics (Rotkreuz, Switzerland). All measurements were batched into a single run. The total imprecision of both assays were below 5%. Galectin-3 was measured using the Human Galectin-3 Quantikine ELISA Kit from R&D (Minneapolis, USA).

#### 2.2.3. Oxidative Stress Biomarkers

Malondialdehyde (MDA) was determined by GC-MS from Thermo Fisher Scientific (CA, USA). After addition of MDA-d 2 as internal standard, derivatization with 2,4-dinitrophenylhydrazine, and chemical ionization in negative mode, the representative ions *m*/*z* 204 (for MDA) and *m*/*z* 206 (for MDA-d 2) were recorded [15].

Colorimetric methods were used to determine total peroxides (TOC), endogenous peroxidase activity (EPA), and the total antioxidant capacity (TAC) purchased from LDN (Labor Diagnostika Nord, Nordhorn, Germany). These assays are based on the reaction between hydrogen peroxide, horseradish peroxidase, and tetramethylbenzidine to give a blue-green colour. After the addition of the stop solution, the colour changes to yellow, which can be measured at 450 nm (reference wavelength 620 nm). A linear standard curve was used for quantification. The intra- and interassay coefficients of variance were less than 5% for all assays [16]. MDA-LDL IgM was measured with the MDA-LDL-IgM ELISA from Omnignostica Ltd. (Höflein/D., Austria), which is standardized on a human monoclonal antibody as described previously [17]. Serum myeloperoxidase (MPO) concentrations were measured with the MPO enzyme-linked immunosorbent assay (ELISA) Kit (Immundiagnostik AG, Bensheim, Germany) according to the manufacturer's instructions. The total imprecision of both ELISA assays was below 7%. Uric acid was determined with the enzymatic colorimetric test from Roche on a COBAS 8000 analyzer.

In the case of the brain-derived neurotrophic factor (BDNF), we used the Quantikine human BDNF immunoassay from R&D systems (Minneapolis, USA). Adrenocorticotropic hormone (ACTH) was determined with the ACTH ELISA from Hölzel Diagnostica (Köln, Germany), and total polyphenols (PPm) were determined according to the manufacturer's instructions with an adapted Folin-Ciocalteu microtitre method from Omnignostica Ltd. (Höflein/D., Austria). In short, the principle of this method is based on the reaction of polyphenols with transition metals. This leads to a dark-coloured complex, which can be measured at 766 nm. Samples are quantified by the use of a standard curve with serial dilutions of a polyphenol standard. The intra- and interassay coefficients of variance were less than 5%.

### 2.3. Statistical Analysis

Statistical analyses were carried out using SPSS 23.0 for Windows 10 (IBM Corp., USA) and Stata 12 (StataCorp, TX, USA). Comparisons between groups were done by the use of the general linear model including body mass index (BMI) as a covariate because heavy workers had significantly higher BMI which itself could be related to oxidative stress and inflammation, as reported previously [18]. Residuals of analyses were stored and tested for deviations from a normal distribution by Kolmogorov-Smirnov tests with Lilliefors-corrected *p* values. In case of a significant deviation, distribution of residuals was inspected, and in case of a skewed distribution, a logarithmic transformation was applied. In all such cases, normality of residuals was obtained after transformation. Homogeneity of variance was tested by Levene's tests. Data are summarized as means within groups and 95% confidence intervals (back-transformed if necessary to the original scale). A similar approach was applied for comparison of 8 h versus 12 h shifts. In this case, the within-subject factor (8 h/12 h shift length) and between-subject factor groups (office versus heavy workers) and their interaction were tested by analysis of variance. Comparisons of 8 h and 12 h shifts within groups were done by linear contrasts. Variables were log-transformed in accordance with the analysis of baseline data. Based on the ratio between ROS and serum antioxidant capacity, the oxidative stress index (OSI) was calculated using the formula (TOC[mmol/L]/TAC[mmol/L] × 100). For all statistical tests, *p* < 0.05 was considered significant.

## 3. Results

An overview about the anthropometric data of the study cohort is given in Table 1. Due to the fact that the BMI was significantly increased in heavy workers versus office workers, all further analyses were corrected with respect to this biometric parameter, because BMI itself was shown to be associated with OS [18].

**Figure 1:**
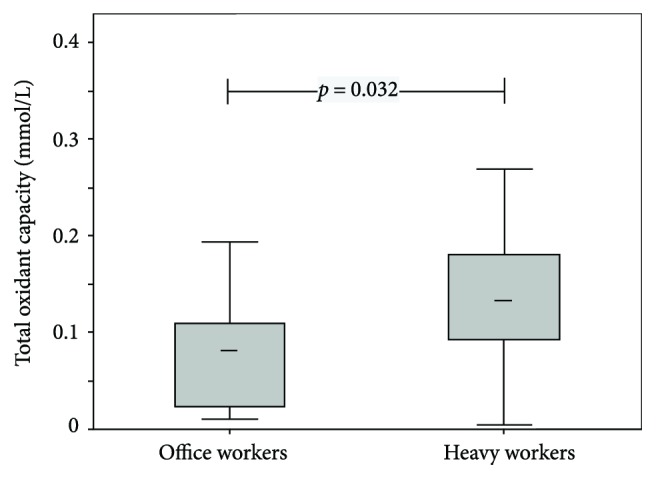
Box plots (medians, interquartile, and nonoutlier ranges) of total oxidant capacity by groups of workers.

**Figure 2:**
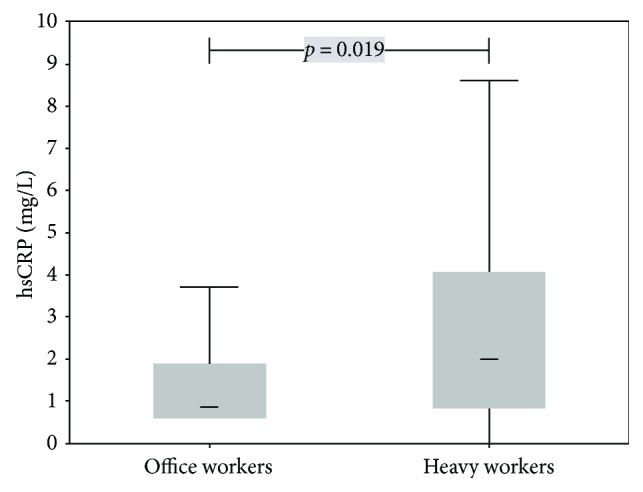
Box plots (medians, interquartile, and nonoutlier ranges) of hsCRP by groups of workers.

**Figure 3:**
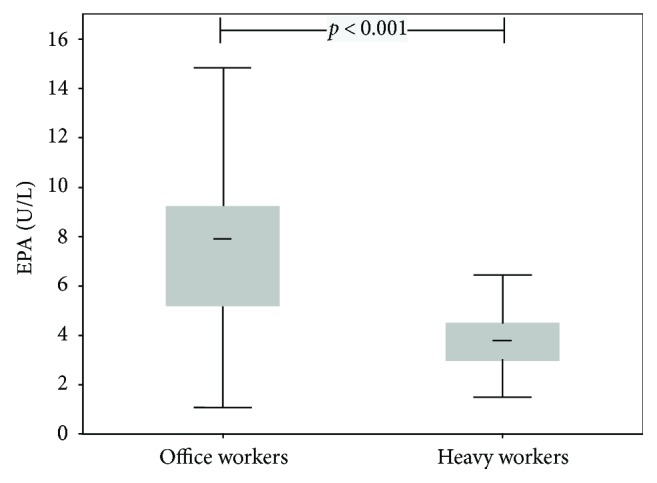
Box plots (medians, interquartile, and nonoutlier ranges) of endogenous peroxidase activity by groups of workers.

**Figure 4:**
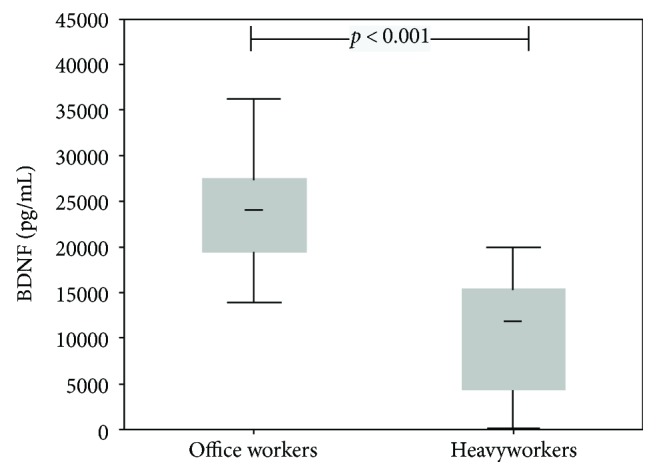
Box plots (medians, interquartile, and nonoutlier ranges) of brain-derived neurotrophic factor by groups of workers.

Heavy workers had significantly increased TAC (*p* < 0.001), TOC (*p* = 0.032), hsCRP (*p* = 0.019), and ACTH (*p* < 0.001) (for details, see Figures 1 and 2 and Table 1) and OSI levels (*p* = 0.011; Table 1). In contrast, EPA (*p* < 0.001), polyphenols (*p* = 0.004), and BDNF (*p* < 0.001) levels were significantly higher in office workers (Figures 3 and 4). Uric acid, MDA, MPO, IL-6, MDA-LDL IgM, and galectin-3 did not differ between the groups (Table 1).

Comparison between 8-hour and 12-hour shifts revealed significant differences exclusively after a 12-hour shift in heavy workers, that is, a significant increased ACTH level (*p* = 0.001), while BDNF was significantly decreased at overtime work (*p* = 0.012) (Table 2). Correlation analysis between oxidative stress biomarkers revealed a significant negative correlation between TAC and EPA in both working groups whereas a positive correlation was found for TAC and uric acid (Table 3). TOC correlated positively with hsCRP in both working groups (*r* = 0.612 and 0.493 in office and heavy workers, resp.). In contrast, IL-6 was correlated to TOC merely in office workers (*r* = 0.462), while the correlation with polyphenols was only significant in heavy workers (*r* = 0.565). Furthermore, TAC showed a highly significant negative correlation with TOC in office workers (*r* = −0.526) (Table 3).

## 4. Discussion

In the present study, we indicated an increased inflammation through raised hsCRP levels at baseline in heavy workers compared to office workers. This was associated with oxidative stress, that is, increased total peroxides and a concomitant decrease of peroxidase activity. In addition, we observed a decrease in polyphenols, although the total antioxidant capacity was increased (Table 1). OSI, which reflects the redox balance between prooxidants and antioxidants, showed significant differences between these two working groups.

This was further related to psychological stress, due to an increase in ACTH and a very low level of BDNF indicating emotional stress (Table 1). In spite of significant differences in several biomarkers between office workers and heavy workers in a slaughterhouse, it must be emphasized that this might even be an underestimation due to the working environment of the latter; that is, low temperatures were previously associated with reduced OS [19, 20].

A stressful working environment may affect the health of employees. Night shifts disrupt the circadian rhythm and increase OS [21]. There can be no doubt that a better understanding of the main stressors in the workplace would be effective in preventing disease and that determination of oxidative stress biomarkers could be helpful in this context [7]. Since reduction of sickness-related absenteeism implies economic benefits, individual health care at the workplace should be given priority. Increased disease risks in workers with demanding jobs have frequently been reported, among others by Ramey et al. [22]. Release of catecholamines and increased blood pressure, along with chronic work-related stress, may lead to cardiovascular diseases. A combination of psychological and physical stress could induce chronic inflammation and subsequent disease [23]. For such reasons, a dietary regimen including antioxidants was suggested [24, 25], but it is not clear if such a strategy is of much help [26], especially if the working conditions otherwise remain unchanged.

Notably, sensitive biomarkers identified these effects, pointing to early development of an imbalance in the redox system. Nevertheless, there were no changes in MDA, one of the end products of lipid peroxidation, MDA-LDL IgM, a biomarker for immune activation, MPO, uric acid, and galectin-3. Although the significant differences seen between occupational groups were fluctuations within “normal” ranges, it must be kept in mind that individuals may be exposed to these changes throughout their working lives. Such mild chronic stress responses over prolonged time periods are in line with our results and were also reported in an animal experiment with increased oxidative stress and consumption of antioxidants, especially in the pancreas. This led to systemic inflammation and contributed to degenerative diseases [27].

It was striking that overtime was accompanied by an almost threefold increase of ACTH and a significant decrease in BDNF in laborers only (Table 2), pointing to a combined impact of a heavy workload and 12 h shift.

Overtime, shift work [23] and extended exposure to occupational and environmental stressors diminish antioxidative capacity, which may elevate the impact of increased production of OS due to a heavy workload [28, 29]. Walker et al. [30] reported that inflammation and alterations of the immune system were associated with altered mood and reduced well-being, thus highlighting the need for improved risk management in the workplace.

We observed a significant correlation between the total antioxidant capacity and uric acid, as has been reported previously [18]. There is also a strong inverse correlation between endogenous peroxidase activity and total antioxidant capacity. The correlation between (hsCRP) and oxidative stress (TOC) underlines the link between inflammation and cellular stress responses (Table 3).

Monitoring with sensitive biomarkers may be advisable, particularly in cases of smoking, obesity, and older age, to counteract an accumulation of stress-related biological changes that could have adverse health effects. Research of oxidative stress under real-life working conditions is a win-win situation for both employers and employees. It could help to tailor health care and counseling for workers, minimizing sickness absenteeism and reducing fluctuation in the workforce.

The small number of manual laborers doing a 12-hour work shift could be a limitation for this study due to insufficient compliance. In addition, the lack of female subjects is a constraint of this work. Therefore, further research in these working groups with a larger collective, including female workers, should be performed.

In conclusion, we found increased oxidative stress and inflammation in manual laborers as compared to office workers. Indications of psychological stress were observed for overtime work in combination with hard physical work. The relationship between antioxidant consumption, oxidative stress, and inflammation was clearly shown in the correlation analysis. These data provide a solid basis for further research on this important subject with a larger collective.

## Figures and Tables

**Table 1:** Baseline characteristics of study participants and results of measurements after an 8-hour work shift.

	Office workers (*n* = 27)	Heavy workers (*n* = 52)	
	Mean (95% confidence interval)		*p*
Age, yrs	38.2 (35.2–41.1)	40.8 (38.5–43.0)	0.175
Body mass index, kg/m^2^	26.1 (24.5–27.6)	28.3 (27.1–29.5)	0.026
hsCRP, mg/L	1.0 (0.7–1.5)	1.7 (1.4–2.2)	0.019
IL-6, pg/mL	1.9 (1.6–2.2)	2.0 (1.7–2.2)	0.220
Uric acid, mg/dL	5.3 (4.8–5.7)	5.6 (5.3–5.9)	0.245
Total antioxidant capacity, mmol/L	1.06 (0.91–1.21)	1.41 (1.29–1.53)	<0.001
Total oxidant capacity, mmol/L	0.08 (0.06–0.11)	0.12 (0.10–0.15)	0.032
Oxidative stress index (OSI), %	5.7 (3.5–8.4)	10.4 (8.2–12.8)	0.011
Endogenous peroxidase activity, U/L	7.2 (6.2–8.4)	3.7 (3.3-4.1)	<0.001
Polyphenols, mmol/L	9.9 (9.7–10.1)	9.5 (9.4–9.7)	0.004
Malondialdehyde, *μ*mol/L	0.74 (0.68–0.80)	0.69 (0.65–0.73)	0.144
Myeloperoxidase, *μ*g/L	61.7 (56.4–67.4)	68.3 (63.7–73.2)	0.078
MDA-LDL-IgM, U/L	184 (149–228)	150 (127–177)	0.141
ST2, ng/mL	14.5 (12.3–16.6)	15.3 (13.7–17.0)	0.521
ACTH, pg/mL	12.0 (9.1–16.0)	27.6 (22.2–34.4)	<0.001
Galectin-3, ng/mL	5.9 (5.0–6.9)	4.8 (4.2–5.4)	0.052
BDNF, pg/mL	22880 (16051–32616)	7417 (5651–9735)	<0.001

*p* values from the general linear model with body mass index included as covariate. hsCRP = high-sensitivity C-reactive protein; IL-6 = interleukin-6; OSI = oxidative stress index; ACTH = adrenocorticotropic hormone; ST2 = suppression of tumorigenicity 2; BDNF = brain-derived neurotrophic factor.

**Table 2:** Comparison of stress and inflammatory biomarkers between 8 and 12 h shifts in office and heavy workers.

	Office workers (*n* = 26)			Heavy workers (*n* = 8)		
	After 8 hours Mean (95% CI)	After 12 hours Mean (95% CI)	*p*	After 8 hour Mean (95% CI)	After 12 hour Mean (95% CI)	*p*
hsCRP, mg/L	1.23 (0.89–1.71)	1.08 (0.82–1.43)	0.311	0.98 (0.54–1.79)	0.84 (0.50–1.39)	0.482
IL-6, pg/mL	1.89 (1.57–2.28)	1.71 (1.51–1.93)	0.350	1.79 (1.27–2.53)	1.97 (1.57–2.46)	0.641
Total antioxidant capacity, mmol/L	1.03 (0.81–1.25)	1.20 (0.94–1.46)	0.236	1.26 (0.91–1.61)	0.98 (0.56–1.40)	0.220
Total oxidant capacity, mmol/L	0.10 (0.07–0.16)	0.10 (0.07–0.15)	0.786	0.05 (0.02–0.10)	0.07 (0.03–0.14)	0.110
Endogenous peroxidase activity, U/L	6.54 (5.12–8.36)	7.23 (6.21–8.43)	0.434	8.00 (5.40–11.86)	8.00 (6.26–10.23)	0.998
Malondialdehyde, *μ*mol/L	0.72 (0.64–0.80)	0.75 (0.65–0.86)	0.610	0.79 (0.64–0.96)	0.76 (0.59–0.98)	0.857
Myeloperoxidase, *μ*mol/L	65.9 (55.0–78.8)	67.5 (59.0–77.4)	0.812	64.5 (46.4–89.7)	56.3 (43.9–72.2)	0.488
Paraoxonase, ng/mL	14.6 (13.1–16.2)	14.2 (12.9–15.5)	0.441	12.7 (10.4–15.4)	11.5 (9.8–13.6)	0.171
ACTH, pg/mL	12.2 (7.7–19.4)	13.6 (10.3–18.0)	0.510	8.8 (3.8–20.5)	24.9 (15.0–41.4)	0.001
BDNF, pg/mL	24030 (21661–26658)	22941 (20777–25330)	0.479	24634 (20370–29790)	17921 (14947–21485)	0.012

*p* values from linear contrasts after analysis of variance with body mass index included as a covariate.

**Table 3:** Spearman correlation coefficients between stress and inflammation biomarkers.

	TAC		TOC		EPA		Polyphenols	
	Office workers (*n* = 27)	Heavy workers (*n* = 52)	Office workers (*n* = 27)	Heavy workers (*n* = 52)	Office workers (*n* = 27)	Heavy workers (*n* = 52)	Office workers (*n* = 27)	Heavy workers (*n* = 52)
hsCRP	−0.181	0.217	0.612^∗∗∗^	0.493^∗∗∗^	0.172	−0.074	−0.132	0.290^∗^
IL-6	−0.219	0.167	0.462^∗^	0.168	0.228	−0.069	−0.098	0.033
Uric acid	0.506^∗∗^	0.516^∗∗∗^	−0.105	−0.001	−0.343	−0.069	−0.083	0.218
TAC			−0.526^∗∗^	−0.058	−0.648^∗∗∗^	−0.633^∗∗∗^	−0.164	0.075
TOC	−0.526^∗∗^	−0.058			0.184	−0.196	0.123	0.565^∗∗∗^
EPA	−0.648^∗∗∗^	−0.633^∗∗∗^	0.184	−0.196			−0.160	−0.292^∗^
Polyphenols	−0.164	0.075	0.123	0.565^∗∗∗^	−0.160	−0.292^∗^		
MPO	0.177	0.152	0.075	0.010	−0.093	−0.097	−0.050	−0.112
Paraoxonase	0.206	0.075	0.106	−0.154	−0.249	−0.221	0.040	−0.016
ACTH	−0.042	0.059	−0.026	−0.096	−0.014	−0.031	0.129	−0.069
BDNF	−0.001	0.048	0.121	−0.003	−0.014	−0.072	0.296	0.007

^∗^ *p* < 0.05, ^∗∗^*p* < 0.01, and ^∗∗∗^*p* < 0.001. hsCRP = high-sensitivity C-reactive protein; IL-6 = interleukin-6; TAC = total antioxidant capacity; TOC = total oxidant capacity; EPA = endogenous peroxidase activity; MPO = myeloperoxidase; ACTH = adrenocorticotropic hormone; BDNF = brain-derived neurotrophic factor.

## Abbreviations

ROS: Reactive oxygen species
OS: Oxidative stress
OSI: Oxidative stress index
TAC: Total antioxidant capacity
TOC: Total oxidant capacity
EPA: Endogenous peroxidase activity
MDA: Malondialdehyde
MPO: Myeloperoxidase
PPm: Total polyphenols
ACTH: Adrenocorticotropic hormone
hsCRP: High-sensitivity c-reactive protein
IL-6: Interleukin-6
BDNF: Brain-derived neurotrophic factor
SD: Standard deviation
IQR: Interquartile range
95% CI: 95% confidence interval
IQRs: Interquartile ranges
CV: Coefficient of variation
ICC: Intraclass correlation coefficient
BMI: Body mass index.
